# *Methanofollis propanolicus* sp. nov., a novel archaeal isolate from a Costa Rican oil well that uses propanol for methane production

**DOI:** 10.1007/s00203-022-03152-w

**Published:** 2022-08-13

**Authors:** Linda Dengler, Julia Meier, Felix Grünberger, Annett Bellack, Reinhard Rachel, Dina Grohmann, Harald Huber

**Affiliations:** 1 Institute of Microbiology and Archaea Centre, University of Regensburg, Universitaetsstrasse 31, 93053 Regensburg, Germany; 2 Electron Microscopy Center, University of Regensburg, Universitaetsstrasse 31, 93053 Regensburg, Germany

**Keywords:** Methanogenesis, Propanol, Costa Rica, Oil well, *Methanomicrobiaceae*

## Abstract

A novel methanogenic strain, CaP3V-MF-L2A^T^, was isolated from an exploratory oil well from Cahuita National Park, Costa Rica. The cells were irregular cocci, 0.8–1.8 μm in diameter, stained Gram-negative and were motile. The strain utilized H_2_/CO_2_, formate and the primary and secondary alcohols 1-propanol and 2-propanol for methanogenesis, but not acetate, methanol, ethanol, 1-butanol or 2-butanol. Acetate was required as carbon source. The novel isolate grew at 25–40 °C, pH 6.0–7.5 and 0–2.5% (w/v) NaCl. 16S rRNA gene sequence analysis revealed that the strain is affiliated to the genus *Methanofollis*. It shows 98.8% sequence similarity to its closest relative *Methanofollis ethanolicus*. The G + C content is 60.1 mol%. Based on the data presented here type strain CaP3V-MF-L2A^T^ (= DSM 113321^T^ = JCM 39176^T^) represents a novel species, *Methanofollis propanolicus* sp. nov.

**Supplementary Information:**

The online version contains supplementary material available at 10.1007/s00203-022-03152-w.

## Introduction

The genus *Methanofollis* (Zellner et al. 1999) represents one out of six genera within the family *Methanomicrobiaceae* (Balch et al. 1979). With the publication of this genus, the former *Methanogenium* species *M. tationis* and *M. liminatans* were transferred to the novel genus *Methanofollis* due to distinct patterns of glycolipids, phosphoglycolipids and amino-phosphoglycolipids among others. Furthermore, *Methanofollis tationis* possesses a pterin that is different from the known methanopterin or sarcinapterin–tatiopterin has not been found in any other methanogen since then (Raemakers-Franken et al. 1991).

Today, six validly described species are assigned to the genus *Methanofollis*. These species were isolated from various aquatic environments with different salinities. *Methanofollis liminatans* was derived from a wastewater reactor in Germany and *Methanofollis tationis* from a solfataric field in Chile. *Methanofollis formosanus* (Wu et al. 2005) and *Methanofollis aquaemaris* (Lai and Chen 2001) were both isolated from a fish pond in Taiwan. *Methanofollis ethanolicus* (Imachi et al. 2009) was discovered in a lotus field, while the recently described *Methanofollis fontis* was isolated from a marine sediment near a cold seep (Chen et al. 2020). With the increasing number of described species, it became clear that the genus’ capability to use primary and secondary alcohols with two or more C-atoms for methane production is a characteristic feature.

CaP3V-MF-L2A^T^ shows a similarity of 98.8% in 16S rRNA gene sequence towards *Methanofollis ethanolicus*; however, their physiological characteristics differ significantly. Strain CaP3V-MF-L2A^T^ cannot grow on ethanol or 1-butanol but utilizes 1-propanol and 2-propanol for methanogenesis. Furthermore, strain CaP3V-MF-L2A^T^ is characterized by a smaller cell size, its motility and a significantly shorter generation time. Therefore, we propose the here described strain CaP3V-MF-L2A^T^ as a novel species, *Methanofollis propanolicus* sp. nov.

## Materials and methods

### Sampling and isolation

Strain CaP3V-MF-L2A^T^ was isolated from an exploratory oil well in Cahuita National Park, located at the southwestern Atlantic coast of Costa Rica (SI Fig. 1). Water samples were taken under sterile and anaerobic conditions in a depth of 20–30 cm sub-watersurface using 100 ml glass bottles.

MS medium, modified from Balch’s medium I (Balch et al. 1979) was used for enrichment and cultivation of strain CaP3V-MF-L2A^T^. This medium contained the following components (l^−1^): 0.45 g NaCl, 5.00 g NaHCO_3_, 0.1 g MgSO_4_ × 7 H_2_O, 0.225 g KH_2_PO_4_ × 3 H_2_O, 0.3 g K_2_HPO_4_ × 3 H_2_O, 0.025 g (NH_4_)_2_SO_4_, 0.06 g CaCl_2_ × 2 H_2_O, 0.002 g (NH_4_)_2_Ni(SO_4_)_2_, 0.002 g FeSO_4_ × 7 H_2_O, 1 ml 0.1% resazurin solution, 1 ml tenfold trace mineral solution and 1 ml tenfold vitamin solution (Huber and Stetter 2006). The medium was prepared according to the standard techniques for anaerobic cultivation (Balch and Wolfe 1976). It was reduced with 0.5 g Na_2_S × 2–3 H_2_O and the pH was adjusted to 6.5 with 1 M HCl. 20 ml medium were aliquoted into 120 ml serum bottles and pressurized with H_2_/CO_2_ (80:20, v/v, 300 kPa) or N_2_/CO_2_ (80:20, v/v, 200 kPa), respectively.

Inoculation was performed with 0.5 ml of original samples in 20 ml medium supplemented with 0.5% acetate (w/v) and H_2_/CO_2_ as gas phase. Initial cell growth with irregular cocci occurred after 1 week of incubation at 37 °C with shaking. After two transfers, single cell isolation was performed using an optical tweezer setup (Huber et al. 1995). For further studies, acetate supplement was reduced from 0.5 to 0.1%. For long-term conservation, cells were anaerobically centrifuged (3000× g, 30 min), resuspended in medium containing 5% DMSO, sealed in glass capillaries and stored over liquid nitrogen in our in-house culture collection. Logarithmic cell cultures were stored at 4 °C for 2–3 months for short-term storage.

### Microscopic techniques

Light microscopy was performed using a phase-contrast and fluorescence microscope (Olympus BX60; Bright Line HC 434/17 excitation filter with a beam splitter 452 nm and long pass filter U-E455). Motility was surveyed at 37 °C under anaerobic conditions using a temperature gradient-forming device (Mora et al. 2014) connected to a phase-contrast microscope (Olympus BX53). Gram-stain was carried out as described previously (Boone and Whitman 1988) using *Escherichia coli* K12 (ATCC^®^ 25922^™^) and *Bacillus atrophaeus* (ATCC^®^ 9372^™^) as reference strains.

For electron microscopy, cells in late exponential growth phase were fixed with 1% glutardialdehyde (final concentration; v/v) for 10 min at room temperature and concentrated by centrifugation (4000× g, 15 min). 10 µl of cell concentrate were placed on hydrophilized 400-mesh carbon-coated copper grids (Plano). Samples on grids were then either negatively stained for 1 min with 2% uranyl acetate (w/v) or shadowed with Pt/C (15° angle; CFE 50; Cressington). Freeze etching was carried out as described previously (Rachel et al. 2002).

Transmission electron micrographs were imaged using a CM12 transmission electron microscope (FEI) operated at 120 keV and equipped with a slow-scan charge-coupled device camera (TEM 0124; TVIPS).

### Phylogenetic analysis

DNA isolation was carried out using the XS-buffer method (xanthogenate-SDS) (Tillett and Neilan 2000) using 2 ml of late exponential cell culture as starting material. The 16S rRNA gene was PCR-amplified using the universal archaeal forward primer 8aF (Eder et al. 1999) and the universal microbial reverse primer 1512uR (Lane 1991). Subsequently, DNA was purified using Wizard^®^ Genomic DNA Purification Kit (Promega) according to the manufacturers’ instructions. Bidirectinoal Sanger sequencing was carried out by LGC Genomics GmbH, Berlin. Sequences were manually revised using 4Peaks 1.8 (Griekspoor and Groothuis 2005) and aligned with 16S rRNA gene sequences of type strains within the family *Methanomicrobiaceae* in MEGAX 10.1.8 (Tamura et al. 2013; Kumar et al. 2018) using the ClustalW alignment (Thompson et al. 1994). An approximately maximum-likelihood tree was constructed applying FastTree 2 (Price et al. 2010) and visualized using iTol (Letunic and Bork 2007).

The G + C content of total DNA was determined by genome sequencing. Therefore, library preparation was performed according to Oxford Nanopore Technologies (ONT, Oxford, United Kingdom) protocol for native barcoding of genomic DNA (with EXP-NBD104 and SQK-LSK109). Sequencing was performed on ONT’s MinION MK1C device (MinKNOW v.20.10.6). After basecalling and demultiplexing using guppy (fast option, qscore cutoff 7, v. 4.2.3), reads were assembled using flye (v. 2.8.2) (Kolmogorov et al. 2019). Finally, the G + C content was calculated from the contig sequences using the Biostrings package in R (Pagès et al. 2022).

### Morphological and physiological characterization

The analysis of physiological parameters such as optimal temperature, pH, NaCl, and substrate specificity were carried out in triplicates. Experiments on the pH spectrum were conducted by adjusting the values at room temperature using anaerobic HCl or NaOH, respectively. To assure constant pH values, pH was controlled over the time of incubation and readjusted if necessary. To study sodium chloride requirements, the salt was added in concentrations of 0–4.0% (w/v) to NaCl-free MS medium. Sodium chloride was tested in intervals of 0.2% between 0 and 1% and in steps of 0.5% ascending from 1%. To determine substrates used for methanogenesis, the following compounds were added to the medium: acetate (17 mM), formate (22 mM), methanol (31 mM), ethanol (22 mM), 1-propanol (17 mM), 1-butanol (14 mM), 2-propanol (17 mM) and 2-butanol (14 mM). Molarities equal 0.1% (v/v) final concentration in the culture medium. In addition, 5 mM ethanol and 0.01% yeast extract were supplemented as described for *Methanofollis ethanolicus* (Imachi et al. 2009). For the calculation of optimal growth, cells were counted every 24 h for 1 week using a Thoma counting chamber (0.02 mm depth).

## Results and discussion

### Phylogenetic analysis

**Fig. 1:**
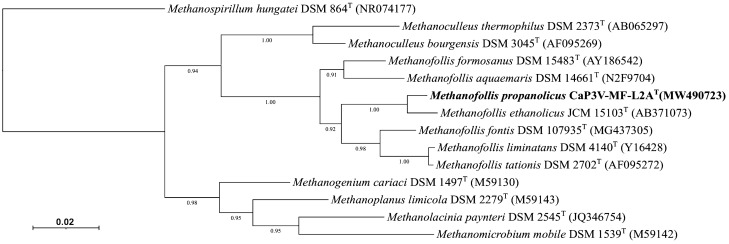
Phylogenetic position of strain CaP3V-MF-L2A^T^ based on 16S rRNA gene sequence of all *Methanofollis* species and the type species of remaining genera within the *Methanomicrobiaceae*. Bootstrap values greater than 90% are displayed. Bar, 2 substitutions per 100 nucleotide positions

Bidirectional sequencing (LGC Genomics) resulted in a 16S rRNA gene sequence fragment of 1364 bp. Phylogenetic analysis showed that strain CaP3V-MF-L2A^T^ is affiliated to the genus *Methanofollis* (Fig. 1). Its closest relative is *Methanofollis ethanolicus* HASU^T^ with a phylogenetic distance of 1.2%. The G + C content was 60.1 mol%.

### Morphological and physiological characterization

**Fig. 2:**
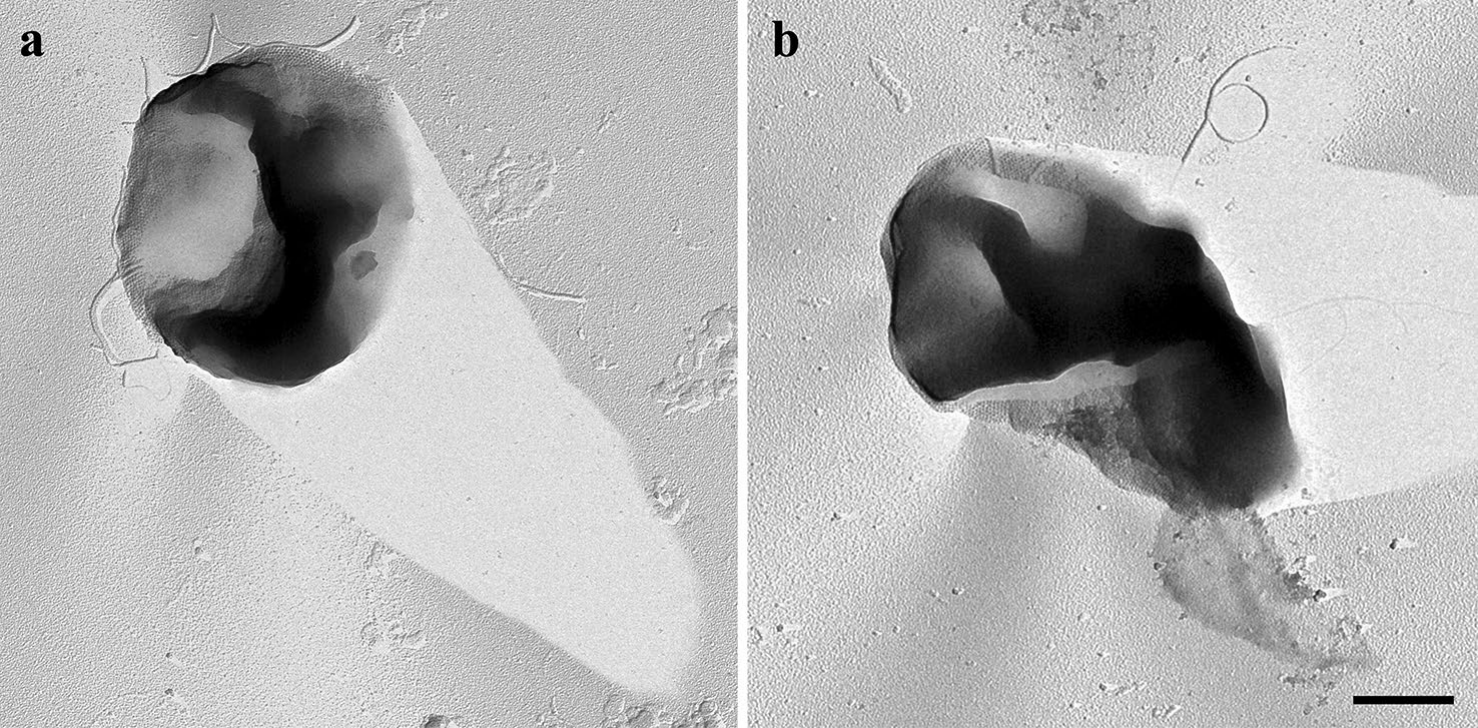
Transmission electron micrograph of Pt/C shadowed cells of strain CaP3V-MF-L2A^T^ show the great variability in cell-shape as well as archaella. Bar, 400 nm

**Fig. 3:**
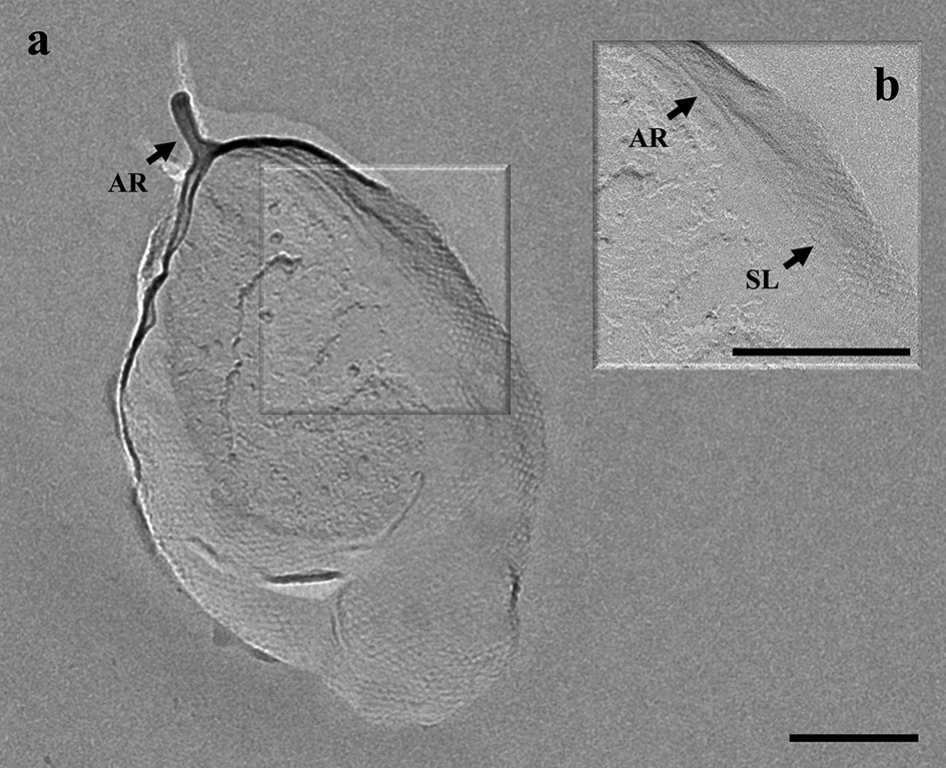
**a** Transmission electron micrograph of freeze-etched cells of strain CaP3V-MF-L2A^T^ indicating **b** proteinaceous S-Layer (SL) and an archaellum (AR). Bars, 200 nm

Cells of strain CaP3V-MF-L2A^T^ showed a greenish autofluorescence of factor F_420_ typically found in methanogens, stained Gram-negative and were motile. In continuous culture, irregularly shaped cocci with a diameter of 0.8–1.8 μm occurred singly or in pairs. Electron microscopy not only revealed an uneven cell surface but also highly variable shape ranging from conical to circularly dent cells (Fig. 2). Cells showed two different types of cell appendages: archaella with a diameter of 12 nm and pili with a diameter of 8 nm. Cells exhibit the typical S-Layer structure of *Methanofollis* species (Fig. 3).

Growth of strain CaP3V-MF-L2A^T^ was detected from 20 to 40 °C, with an optimal growth temperature of 37 °C. A pH of 6.0–7.5 supported cell growth, while levels below or above did not. The optimal pH was determined at pH 6.5–7.0. Strain CaP3V-MF-L2A^T^ tolerated sodium chloride concentrations from 0 to 2.5% (w/v), while the optimal range was 0.2–1.5%.

Strain CaP3V-MF-L2A^T^ used H_2_/CO_2_, formate, 1-propanol, and 2-propanol as energy source, while acetate was required as a carbon source. This substrate spectrum differed from all other described species of the genus *Methanofollis* (Table 1). Yeast extract had a stimulating effect on cell growth as described for *M. ethanolicus* and *M. liminatans*. Acetate as well as the primary and secondary alcohols methanol, ethanol, 1-butanol, 2-butanol, and cyclopentanol did not support cell growth when used alone or in combination with acetate or yeast extract. This was even true for the substrate combination of 5 mM ethanol and 0.01% yeast extract with an incubation time of up to 3 months (which works for *Methanofollis ethanolicus)*. The doubling time of strain CaP3V-MF-L2A^T^ under optimal physiological conditions was 16 h.

**Table 1:** Characteristics of strain CaP3V-MF-L2A^T^ compared to all other validly described species of the genus *Methanofollis*

	1	2	3	4	5	6	7
Cell morphology	Irregular cocci	Irregular cocci	Irregular cocci	Irregular cocci	Irregular cocci	Irregular cocci/ring shaped	Irregular cocci
Cell diameter (μm)	0.8–1.8	1.5–3	1.2–2	2–3	1.5–2	0.4–0.5	0.8–1.2
G + C content (mol%)	60.1	54	59.1	60.9	58.4	60	59.5
Temperature (°C) optimum (range)	37 (25–40)	37–40 (25–45)	37 (20 –43)	37 (15–40)	37 (20–42)	40 (25–44)	37 (20–40)
pH optimum (range)	6.5–7.0 (6.0–7.5)	7 (6.3–8.8)	6.5 (6.3–8)	7 (6.5–7.5)	6.6 (5.6–7.3)	7	5.9–8.2 (6.7–7.0)
NaCl (%) optimum (range)	0.2–1.5 (0–2.5)	0.8–1.2 (0–7)	0–6 (0.5)	(0–2.5)	3 (0–4)	0 (0–3.5)	0.17 (0–0.85)
Generation time (h)	16	12	13	72	20	7.5	21
Substrates for methanogenesis							
H_2_/CO_2_	+	+	+	+	+	+	+
Formate	+	+	+	+	+	+	+
Ethanol	–	–	–	+	–	–	–
1-Propanol	+	–	ND	+	ND	–	–
1-Butanol	–	–	ND	+	ND	–	–
2-Propanol	+	–	–	–	–	+	–
2-Butanol	–	–	–	–	–	+	–
Cyclopentanol	–	–	–	–	–	+	–
Growth requirement							
Acetate	+ r^a^	+ r	+ s	+ s	+ s	+ s	+ s
Yeast extract	+ s	+ r	+ s	+ r	+ s	+ r	ND
Motility	+	–	–	–	–	+	–

Strains: 1, CaP3V-MF-L2A^T^ (data from the present study); 2, *Methanofollis tationis* OCM 159^T^ (Zabel et al. 1984); 3*, Methanofollis aquaemaris* N2F9704^T^ (Lai and Chen 2001); 4*, Methanofollis ethanolicus* HASU^T^ (Imachi et al. 2009); 5, *Methanofollis formosanus* ML15^T^ (Wu et al. 2005); 6, *Methanofollis liminatans* BM1^T^ (Zellner et al. 1990, 1999; Zellner and Boone 2001); 7, *Methanofollis fontis* FWC-SCC2^T^ (Chen et al. 2020)

+  positive, – negative; *ND* not determined, *s* stimulates growth, *r* required

^a^Acetate is required as carbon source on substrates other than 1-propanol and 2-propanol

The ancient oil well, where strain CaP3V-MF-L2A^T^ was isolated from, represents an open pond with plenty of organic import (e.g., leaves from surrounding fauna). This explains the availability of substrates, such as acetate or propanol. In the natural environment, propanol derives from anaerobic microbial degradation processes. 2-propanol is known to be produced by some saccharolytic *Clostridia* by the reduction of acetone (Langlykke et al. 1937; Kutzenok and Aschner 1952; George et al. 1983).

## Taxonomic conclusion

Based on phylogenetic, morphological, and physiological characteristics, strain CaP3V-MF-L2A^T^ is considered to display a novel species within the genus *Methanofollis* (Table 1).

### Description of *Methanofollis propanolicus* sp. nov.

*Methanofollis propanolicus* sp. nov. (pro.pa.no´li.cus. N.L. n. propanol; L. suf. -icus -a -um suffix used with various meanings; N.L. masc. adj. propanolicus) regarding to propanol, based on the substrate propanol, which can be metabolized by this species.

Cells are irregular cocci, motile, 0.8–1.8 μm in diameter, and occur as single cells or in pairs. Occurrence of at least two types of cell appendages. Strictly anaerobic. Temperature range for growth 25 °C–40 °C (optimum, 37 °C). Sodium chloride range for growth 0–2.5% (w/v) (optimum, 0.2–1.5%). pH range for growth 6.0–7.7 (optimum, pH 6.5–7.0). Doubling time is 16 h. H_2_/CO_2_, formate, 1-propanol and 2-propanol used for methanogenesis, addition of 0.1% acetate is crucial for growth on substrates different from 1-propanol or 2-propanol. G + C content of DNA is 60.1 mol%. Closely related to *Methanofollis ethanolicus* JCM15103^T^ (98.8% 16S rRNA gene sequence similarity).

The type strain is CaP3V-MF-L2A^T^ (= DSM 113321^ T^ = JCM 39176^ T^), isolated from an oil well in the Cahuita National Park, Costa Rica.

The GenBank/EMBL/DDBJ accession number for the 16S rRNA gene sequence of strain CaP3V-MF-L2A^T^ is MW490723.

## Supplementary Information

Below is the link to the electronic supplementary material.

SI Fig 1: Ancient oil well in the Cahuita National Park, Costa Rica, original sampling site of strain CaP3V-MF-L2AT Supplementary file1 (JPG 68748 KB)
